# Protective Effect of *Simplicillium* sp. Ethyl Acetate Extract against High Glucose-Induced Oxidative Stress in HUVECs

**DOI:** 10.1155/2020/5172765

**Published:** 2020-08-15

**Authors:** Ting-Ting Tian, Qi-Rui Li, Shi-Quan Gan, Chu-Rui Chang, Xiang-Chun Shen

**Affiliations:** ^1^The Key Laboratory of Optimal Utilization of Natural Medicine Resources, School of Pharmaceutical Sciences, Guizhou Medical University, University Town, Guiyang, Guizhou, China; ^2^The High Educational Key Laboratory of Guizhou Province for Natural Medicinal Pharmacology and Druggability, School of Pharmaceutical Sciences, Guizhou Medical University, University Town, Guiyang, Guizhou, China; ^3^The Union Key Laboratory of Guiyang City-Guizhou Medical University, School of Pharmaceutical Sciences, Guizhou Medical University, University Town, Guiyang, Guizhou, China; ^4^The State Key Laboratory of Functions and Applications of Medicinal Plants, Guizhou Medical University, University Town, Guiyang, Guizhou, China

## Abstract

This study aimed at investigating the cytoprotective effect of an ethyl acetate extract of insect fungi against high glucose- (HG-) induced oxidative damage in human umbilical vein endothelial cells (HUVECs). An insect fungus strain termed CH180672 (CH) was found for protecting HUVECs from HG-induced damage. In this study, CH was identified as *Simplicillium* sp. based on a phylogenetic analysis of ITS‐rDNA sequences. Ethyl acetate extract (EtOAc) of this strain (CH) was subjected to the following experiments. Cell viability was examined with the MTT method. To evaluate the protection of CH, intracellular reactive oxygen species (ROS), malondialdehyde (MDA) levels, and the activities of antioxidant enzymes were measured and the expression of oxidation-associated proteins was assessed. In the current study, it has been found that CH can increase the survival rate of HUVECs induced by HG. Additionally, we found that HG-induced nuclear factor-erythroid 2-related factor 2 (Nrf2) and heme oxygenase 1 (HO-1) signal decreased and increased the intracellular ROS and MDA generation in HUVECs. However, CH treatment strongly promoted the translocation of Nrf2 and its transregulation on HO-1 and ultimately inhibited the high level of ROS and MDA induced by HG. The regulatory ability of CH was similar to Nrf2 agonist bardoxolone, while the effect was abolished by ML385, suggesting that Nrf2 mediated the inhibition of CH on HG-induced oxidative stress in HUVECs. Taken together, CH can improve HG-induced oxidative damage of HUVECs, and its mechanism may be related to the regulation of the Nrf2/HO-1 pathway.

## 1. Introduction

Diabetes mellitus (DM) is an endocrine and metabolic disorder characterized by impaired insulin secretion and increased blood glucose level [1]. Hyperglycemia causes many serious complications such as retinopathy, neuropathy, and nephropathy in diabetic patients [2]. It is notable that hyperglycemia causes a significant increase in reactive oxygen species (ROS) in endothelial cells, leading to cell injury and subsequent cell death [3]. It has been reported that ROS-mediated vascular stress can maintain glucose normalization in DM animals and in cultured endothelial cells. Long-term maintenance of oxidative stress results in the overactivation of pathways closely associated with DM and diabetic complications [4]. Oxidative stress is the initial link and pathological factor of endothelial damage and microvascular diseases. Reducing excessive ROS is crucial for the prevention and treatment of DM complications, especially in endothelial dysfunction and injury.

Nuclear factor-erythroid 2-related factor 2 (Nrf2) is an important antioxidant-regulated transcription factor. Under normal conditions, Nrf2 is constitutively expressed and remains inactive in the cytosol [5]. In response to oxidative stress, Nrf2 is released from its interacting protein and translocates to the nucleus [6, 7], thereby initiating the transcription of substream antioxidant genes, including superoxide dismutase (SOD), catalase, heme-oxygenase 1 (HO-1), and NAD(P)H: quinone oxidoreductase 1 (NQO1) to inactivate the stressors and restore homeostasis [8, 9]. Therefore, the activation of Nrf2 is a key target for the treatment of diabetic microvascular complications and developing new drugs.

*Simplicillium* belongs to insect fungi which are ancient resources in Chinese medicine. It has been reported that insect fungi have several beneficial biological activities. It has been used for multiple medicinal purposes such as hypoglycemic, hypolipidemic, anti-inflammatory, antitumor, antimetastatic, and antioxidant effects [10–13]. In addition, the mycelium of insect fungi has been reported to show strong effects on specific markers of oxidative stress, and its protective effects on heart and blood vessels have been observed in the I/R mouse model [14]. *Simplicillium* sp. was obtained from insect fungi in Guizhou. The constituent of *Simplicillium* sp. has never been tested for pharmacological activities. Therefore, we considered whether the *Simplicillium* sp. (strain CH180672) can reduce HG-induced HUVEC damage and then explore its mechanism.

## 2. Materials and Methods

### 2.1. Materials

3-(4,5-Dimethylthiazol-2-yl)-2,5-diphenyl tetrazolium bromide (MTT), endothelial cell medium (ECM), fetal bovine serum (FBS), penicillin/streptomycin, endothelial cell growth factor, and trypsin neutralization solution were purchased from ScienCell (San Diego, California, USA). 0.25% trypsin-EDTA was purchased from Gibco (Grand Island, NY, USA). 2′,7′-Dichlorofluorescein diacetate DCF-DA was purchased from Beyotime. Primary antibodies Nrf2, HO-1, NQO1, GAPDH, and Lamin B1 were from Proteintech (Wuhan, China). Secondary antibodies including anti-mouse and anti-rabbit were purchased from Bioworld Technology (Minnesota, USA). Bardoxolone and ML385 were purchased from MedChemExpress (Shanghai, China). The specific Nrf2 small-interfering RNA (siRNA) and negative control of siRNA were purchased from GenePharma (Shanghai, China). Enhanced chemiluminescence (ECL) western blotting detection solution was obtained from Beyotime (Shanghai, China).

### 2.2. Culture and Extraction of Insect Fungi

Insect fungi samples were collected in Guizhou province. Briefly, the fungi were isolated with the method of single-spore isolation. The strains were seeded on potato dextrose agar (PDA) medium and cultured in an incubator at 28°C for 10 days. Agar plugs were cut into small pieces (about 0.5 × 0.5 × 0.5 cm^3^) under aseptic conditions, and 5 pieces for each were used to inoculate three Erlenmeyer flasks (250 mL), each containing 120 mL of media (NaNO_3_ 3 g/L, K_2_HPO4 1 g/L, MgSO_4_·7H_2_O 0.5 g/L, KCl 0.5 g/L, FeSO_4_·7H_2_O 0.01 g/L, D(+)-sucrose 30 g/L, and agar 15 g/L; the final pH of the media was adjusted to 6.5 and the media were sterilized by autoclaving). Three flasks of the inoculated media were incubated at 28°C on a rotary shaker at 120 rpm for five days and then disposed for 15 days. After incubation, the mycelium and fermentation broth were extracted with ethyl acetate (EtOAc), and the extracts were concentrated using a rotary evaporator in a vacuum at 40°C and then freeze-dried at −70°C. The powdered samples (51.2 mg) were dissolved in dimethyl sulfoxide (DMSO) and stored at −20°C until requirement.

### 2.3. DNA Extraction and ITS Sequencing of the *Simplicillium* sp. Isolate

The total DNA of the CH was extracted using a Fungal DNA Kit following the manufacturer's instructions (Fungal DNA Kit; OMEGA Bio-Tek; USA). ITS‐rDNA gene was amplified with primers ITS1/ITS4 (ITS1: 5′‐TCCGTAGGTGAACCTGCGG‐3′ and ITS4: 5′‐TCCTCCGCTTATTGATATGC‐3′). Each PCR was carried out in 25 *μ*L, containing 12.5 *μ*L 2 × HiFi Taq PCR StarMix buffer (GenStar, China), 1 *μ*L of each primer, 1 *μ*L of total DNA, and 9.5 *μ*L of ddH_2_O. The reaction was then performed using the following reaction cycles: initial denaturation at 94°C for 3 min, followed by 35 cycles of denaturation at 94°C for 30 s, annealing at 55°C for 30 s, and extension at 72°C for 45 s, and then a final extension phase at 72°C for 10 min. PCR products were visualized by 1.0% agarose gel electrophoresis, stained with Golden View in 1 × TBE buffer, and photographed under UV light. Amplified PCR products were sent to the Beijing Genomics Institute for implementing one‐way sequencing with PCR primers.

### 2.4. Cell Culture and Screening of Active Strains

HUVECs were cultured in complete medium (ECM, 5% fetal bovine serum, 1% P/S, 1% ECGS) and were incubated in 5% CO_2_ at 37°C. Screening of active strains was determined using the 3-(4,5)-dimethylthiahiazo (-z-y1)-3,5-diphenytetrazoliumromide (MTT) assay. 1 × 10^5^ cells were seeded in 96-well plates and cotreated with different strains and HG (35 mM) for 24 h, MTT solution was added to each well, and the samples were incubated for 4 hours. The formazan was dissolved by DMSO, and then the optical density was taken at 490 nm using a microplate reader (BioTek Instruments, Vermont, USA).

### 2.5. Intracellular ROS Analysis

The ROS was determined by a commercial ROS assay kit, using the fluorescent probe DCFH-DA. HUVECs were incubated in 24-well plates with HG (35 mM) in the presence or absence of CH for 24 h, and then cells were washed twice with serum-free medium and stained with 40 *µ*L diluted DCFH-DA at 37°C for 45 min. Intracellular fluorescence was measured at excitation and emission wavelengths of 488 and 525 nm, respectively, and pictures were taken with a fluorescence microscope.

### 2.6. Detection of Malondialdehyde

The malondialdehyde content (MDA) was measured by the thiobarbituric acid (TBA) method. HUVECs were exposed to HG with or without CH. After 24 hours of administration, cells were washed twice with cold PBS and lysed in RIPA buffer, and released proteins were collected. The remaining steps are carried out according to the manufacturer's instructions.

### 2.7. Measurement of Antioxidant Enzyme Activities

HUVECs were cultured in 25 cm^3^ cell flasks and treated with CH and HG for 24 h, the supernatant was collected, and cells were washed twice with ice-cold PBS and lysed in RIPA buffer. Protein concentrations were determined using the BCA assay. SOD, GSH, and CAT activities were detected using WST-8, Microenzyme label and Visible light methods, especially, according to the manufacturer's instructions. Experimental assay kits were purchased from Nanjing Jiancheng Institute of Bioengineering, and the experimental steps were performed according to the manufacturer's instruction.

### 2.8. Nuclear and Cytoplasmic Extraction

The cytoplasmic and nuclear fractions were separated with extraction reagents. HUVECs were cultured in 25 cm^3^ cell flasks, treated with CH and HG for 24 h, and then washed thrice with cold PBS. Nuclear protein was extracted using a nuclear and cytoplasmic extraction kit according to the manufacturer's instructions. The protein concentration was determined by a BCA method. The protein samples were denatured in a sodium dodecyl sulfate (SDS) sample buffer at 100°C for 5 min and then stored at −80°C until further experimentation.

### 2.9. siRNA Transfection

The manufacturer's instructions were followed. The special sequences of Nrf2-siRNA were as follows: sense: 5′-CCAGAACACUCAGUGGAAUTT-3′ and antisense: 5′-AUUCCACUGAGUGUUCUGGTT-3'. For each transfection, the medium needs to be changed to 1.5% 1640 1 hour in advance, and 22.5 *µ*l siRNA was added to 1125 *µ*l serum-free medium and 1125 *µ*l serum-free medium containing 22.5 *µ*l Lip2000 transfection reagent is gently mixed. After incubating at 37°C for 5 minutes, the complex was added to the cells in 2 ml of transfection medium, and the mixture was incubated at 37°C for 6 hours. The transfection medium was then replaced with normal medium, and the cells were cultured for 24 hours.

### 2.10. Western Blot Analysis

HUVECs were cultured in 25 cm^3^ cell flasks, treated with CH and HG for 24 h, washed twice with ice-cold PBS, and lysed in RIPA buffer. Protein concentrations were determined using the BCA assay. Western blotting was conducted by loading 30 *µ*g/mL of protein into the wells of 10% SDS polyacrylamide gels. After separation, proteins were transferred to polyvinylidene difluoride (PVDF) membranes, which were then blocked for 2 h at room temperature in 5% BSA, washed three times with 1 × TBST, and incubated with primary antibodies, anti-Nrf2 (1 : 1000; Proteintech), anti-HO-1 (1 : 1000; Proteintech), anti-NQO1 (1 : 1000; Proteintech), GAPDH (1 : 5000; Proteintech), and Lamin B1 (1 : 5000; Proteintech) overnight at 4°C. Then membranes were washed three times with 1 × TBST for 10 min and incubated with the corresponding secondary antibodies for 1 h at room temperature and then washed three times with 1 × TBST for 10 min. Finally, protein bands were detected by ECL and quantified densitometrically with ImageJ software (ChemiDoc XRS, American Bio-Rad).

### 2.11. Statistical Analysis

Data were subjected to analysis of variance (ANOVA) using GraphPad Prism® 6.0 software (La Jolla, CA, USA). Statistical analysis was undertaken using ANOVA and *t*-test for 2 groups and one-way ANOVA for multiple comparisons. Data are expressed as the mean ± SEM. A value of *P* < 0.05 was considered to be statistically significant. Data from *in vitro* studies were derived from at least 3 independent experiments.

## 3. Results and Discussion

### 3.1. Screening Active Strains from Insect Fungi

**Figure 1:**
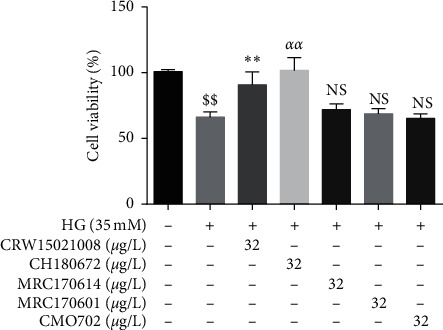
Screening of active strains. Screening of active strains by MTT assay. HUVECs were cotreated with high glucose (HG 35 mM) with or without fungal extract for 24 h, and cell viability was measured by MTT. Data were represented as the mean ± SEM (*n* = 3). ^$$^*P* < 0.01 vs. control group; ^*∗∗*/*αα*^*P* < 0.01 vs. HG group; ^N.S^*P* > 0.05 vs. HG group.

It is well known that endothelial dysfunction is a key pathological progress of numerous cardiovascular diseases. Therefore, we used HG to establish a HUVEC injury model to screen active strains. The same concentration (32 μg/L) EtOAc extracts from 5 different strains of insect fungi was supplied into HG-treated HUVECs. Results revealed that cell viability percentage was significantly enhanced when treated with HG (35 mM) as compared to the control group. Cell viability percentage of EtOAc extracts from CH180672 and CRW15021008 was significantly increased as compared to the HG-treated group (Figure 1). It indicated that extracts of CH180672 and CRW15021008 have protective effects on HG-induced HUVEC injury. CH180672 could significantly ameliorate the HUVEC injury which was identified as *Simplicillium* sp. by blasting their ITS sequences on NCBI. In the following experiments, we will focus on CH180672.

### 3.2. Sequencing and Phylogenetic Analysis of ITS Genes

**Figure 2:**
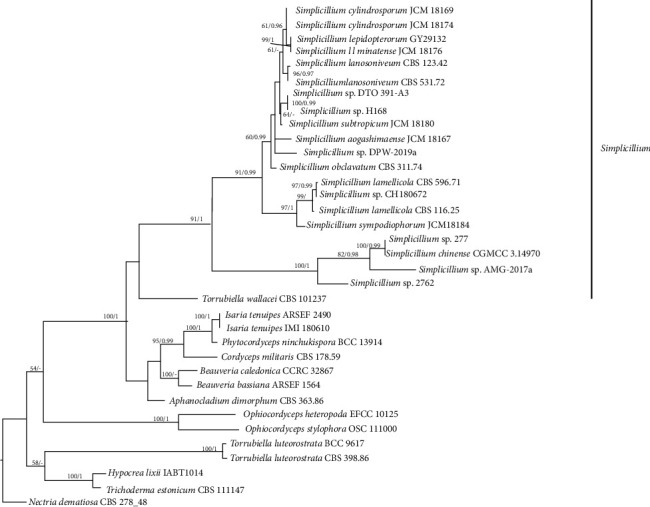
RAxML tree for *Simplicillium* and related genera based on a dataset of ITS‐rDNA gene segment sequences. Bayesian posterior probabilities >0.95 and bootstrap support values for maximum likelihood (ML) higher than >50% are marked above the nodes; an en-dash (“–”) indicates a value <0.95 (PP) or <50% (BS). Strain numbers are noted after the species names. Our new sequence is shown in bold. The tree is rooted via the outgroup *Nectria dematiosa* CBS 278.48.

Here we identified CH180672 as a species of *Simplicillium* sp. based on phylogenetic analysis.  Figure 2 shows that our strain (CH180672) falls in the genus *Simplicillium* with high support (91/1). Strain CH180672 showed a close relationship with *Simplicillium lamellicola* (F.E.V. Sm.) Zare & W. Gams. The *Simplicillium* sp. belongs to insect fungi and is widely used in traditional Chinese herb from ancient times in the treatment of heart and kidney diseases, hyperlipidemia, and hyperglycemia [15, 16].

### 3.3. CH Ameliorated HG-Induced HUVEC Injury

**Figure 3:**
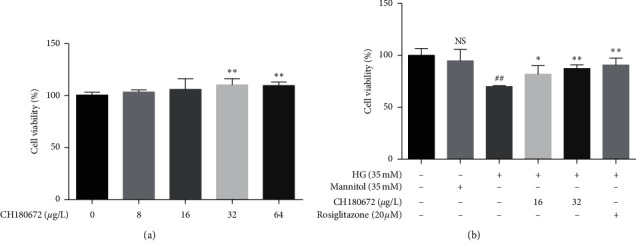
CH ameliorated HG-induced cell damage in HUVECs. (a) HUVECs were stimulated with different concentrations of CH (0, 8, 16, 32, and 64 *μ*g/L) for 24 hours. And then the cell viability was measured using MTT assay. (b) Measurement of CH cell viability by MTT assay. HUVECs were cotreated with high glucose (35 mM HG) with or without CH for 24 h, mannitol (35 mM) was used as an osmotic control group, and rosiglitazone (20 *µ*M) was used as a positive group. Data were represented as the mean ± SEM (*n* = 3). ^N.S^*P* > 0.05 vs. control group; ^##^*P* < 0.01 vs. control group; ^*∗*^*P* < 0.05 vs. HG group; ^*∗∗*^*P* < 0.01 vs. HG group.

Firstly, we examined the effect of CH on cell viability in HUVECs. The results of MTT assay showed that CH concentration of 16 and 32 *μ*g/L did not reduce the viability of HUVECs. It indicated that 16 and 32 *μ*g/L CH were nontoxic to HUVECs (Figure 3(a)). We then investigated the effect of CH on HG-induced HUVECs, at both low and high doses. We initially exposed HUVECs to HG (35 mM), and cell damage percentage caused by 35 mM HG was 25% which is in the range of 20%–30%. It indicated that the cell damage model was established successfully. Cell viability percentage was significantly enhanced when treated with 16 *µ*g/L CH (*P* < 0.05) and 32 *µ*g/L CH (*P* < 0.01), in which pretreatment with CH obviously increased the cell viability of HUVECs in a dose-dependent manner (Figure 3(b)). Our results indicated that CH can ameliorate HG-induced endothelial damage.

### 3.4. CH Reduced the Generation of ROS in HUVECs Induced by HG

**Figure 4:**
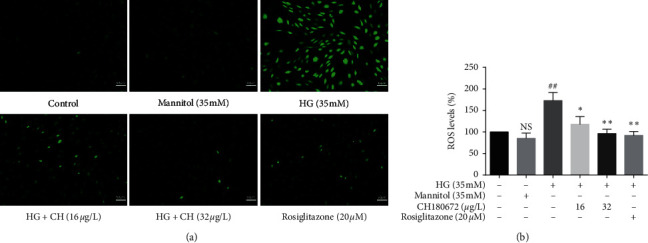
Effect of CH on intracellular reactive oxygen species (ROS) production in HG-induced HUVECs. (a) Intracellular ROS levels in HUVECs were assessed DCFH-DA probe after treated with high glucose (HG, 35 mM) with or without CH for 24 h. HUVECs were loaded with DCFH-DA in the dark at 37°C for 40 min and then pictures were collected with a fluorescence microscope (magnification 200×). (b) Intracellular fluorescence was measured using a fluorescence microplate at excitation and emission wavelengths of 488 and 525 nm. Results were represented as the mean ± SEM (*n* = 3). ^N.S^*P* > 0.05 vs. control group; ^##^*P* < 0.01 vs. control group; ^*∗*^*P* < 0.05 vs. HG group; ^*∗∗*^*P* < 0.01 vs. HG group.

As oxidative stress is crucial for the HG-induced cell injury, it has been reported that decreasing oxidative stress by lowering ROS production is crucial in the management of diabetes complications [17]. We next detected the ROS level in HUVECs. HG treatment produced tremendous fluorescence in HUVECs, suggesting HG-induced oxidative stress to cause endothelial injury. Cotreatment with HG and CH at low and high doses can inhibit increased ROS level induced by HG (Figures 4(a) and 4(b)). Our results proved that CH reduced intracellular ROS accumulation caused by HG.

### 3.5. CH Reduced the Malondialdehyde (MDA) in HUVECs Induced by HG

**Figure 5:**
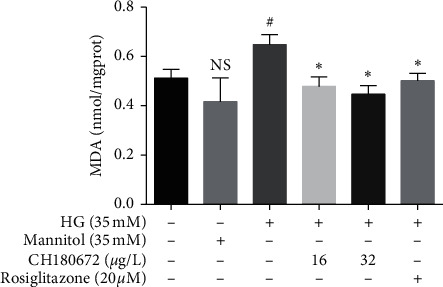
Effect of CH on malondialdehyde (MDA) levels in HG-induced HUVECs. HUVECs were treated with high glucose (HG, 35 mM) with or without CH for 24 h. MDA levels were using a TBA method. Results were represented as the mean ± SEM (*n* = 3). ^N.S^*P* > 0.05 vs. control group; ^#^*P* < 0.05 vs. control group; ^*∗*^*P* < 0.05 vs. HG group.

ROS can attack unsaturated fatty acids in biofilms and form lipid peroxides. The metabolite malondialdehyde (MDA) is a harmful peroxide. MDA content can directly reflect the degree of lipid peroxidation in cells and indirectly reflect the degree of cell damage. HG increases nonenzymatic glycosylation and results in ROS overproduction, lipid peroxidation, and lipid metabolic disorder. We measured relative rates of lipid peroxidation by measuring levels of MDA, the product of polyunsaturated fatty acid, and related ester breakdown [18].  Figure 5 shows that the level of MDA in the HG group increased significantly compared with the normal group. When pretreated with CH, the level of intracellular MDA was significantly reduced. These data supported that CH may reduce cell damage exposed to HG.

### 3.6. CH Restored the SOD, CAT, and GSH Levels in HG-Induced HUVECs

**Figure 6:**
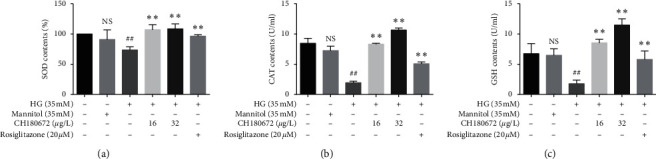
Effect of CH on antioxidative enzyme activity in HG-induced HUVECs. (a) After 24 h of CH treatment for HG-induced HUVECs, SOD activity was measured. (b) CH reversed the CAT content inhibited by HG treatment for 24 h. (c) GSH level was measured using Microenzyme label method after 24 h of CH treatment with HG (35 mM). Data were represented as the mean ± SEM (*n* = 3). ^N.S^*P* > 0.05 vs. control group; ^##^*P* < 0.01 vs. control group; ^*∗∗*^*P* < 0.01 vs. HG group.

After HUVECs were exposed to HG, the activities of SOD and CAT were significantly decreased as well as GSH level. CH could inhibit the decrease of SOD, CAT, and GSH levels (Figures 6(a)–6(c)). These results indicated that CH protected oxidative stress damage of HUVECs induced by HG through cellular antioxidation system to control redox balance. Antioxidant enzymes such as superoxide dismutase (SOD) and catalase (CAT) and meanwhile reduced glutathione (GSH) act as defense systems.

However, both oxidant and antioxidant balance are disrupted and cells are damaged by oxidative stress [19]. Our experimental results indicate that HG leads to reduced activity of the endogenous antioxidant enzyme SOD, depleted GSH levels, and decreased cellular CAT while the presence of CH significantly improved their production.

### 3.7. CH Modulated Nrf2 Translocation and Expression in HG-Induced HUVECs

**Figure 7:**
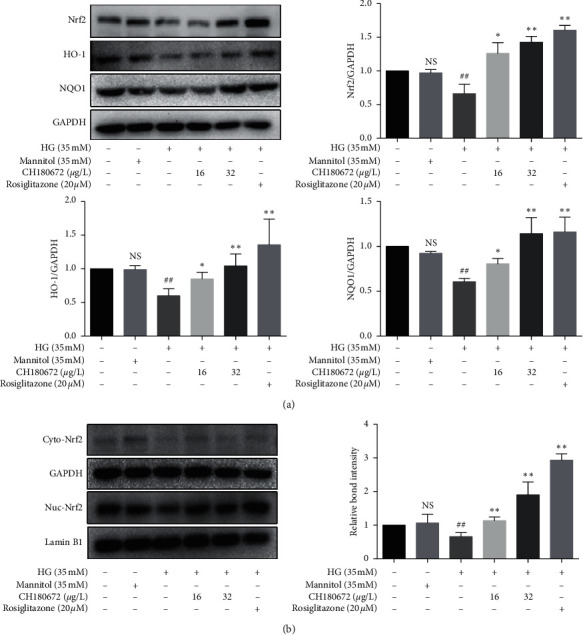
CH increases the Nrf2 protein level in HG-induced HUVECs. (a) Western blot analysis of Nrf2, HO-1, and NQO1 protein level in the whole cell lysate after pretreatment with CH for 2 h and exposed to HG for 24 h. (b) Western blot analysis of the Nrf2 protein level in the nucleus and cytoplasm. 24 h of HG treatment induced the nuclear accumulation of Nrf2, and CH treatment enhanced the nuclear accumulation of Nrf2 activated by HG. Data were represented as the mean ± SEM (*n* = 3). ^N.S^*P* > 0.05 vs. control group; ^##^*P* < 0.01 vs. control group; ^*∗*^*P* < 0.05 vs. HG group; ^*∗∗*^*P* < 0.01 vs. HG group.

In the previous study, it was found that cotreatment of HG and CH could quench the overproduction of ROS and MDA induced by HG. Next, the effect of CH on HG-induced HUVECs was investigated at the protein level. Nrf2 is a basic leucine zipper protein that under normal conditions is retained in the cytoplasm [20]. Oxidative stress promotes Nrf2 to translocate into the nucleus where it binds to the AREs in the promoter region of many antioxidant genes such as HO-1 and NAD(P)H dehydrogenase, quinone 1 (NQO1) [21, 22]. Growing evidence indicates that an acute increase in glucose is accompanied by a reduction in endogenous antioxidant genes that may result in endothelial cell dysfunction [23, 24]. In order to explore whether CH plays a protective role through the Nrf2 signaling pathway, we determined whether the increased ROS level may be related to the activation of the antioxidant Nrf2 signal. Western blot analysis found that the protein expression of Nrf2 in the HG model was reduced but increased in HUVECs treated with CH. Similar results were obtained in HO-1 and NQO1 expression by western blot (Figure 7(a)). To further ascertain whether CH treatment altered the Nrf2 nucleus translocation, we isolated nuclear and cytoplasmic proteins of HUVECs in different groups and found that 24 h treatment with CH upregulated the protein abundance of nuclear Nrf2, indicating that Nrf2 was translocated to the nucleus (Figure 7(b)). Our results indicated that CH attenuated oxidative stress and enhanced the nuclear translocation of Nrf2 to stimulate the Nrf2 pathway.

### 3.8. CH Ameliorated High Glucose-Induced Endothelial Damage *via* Nrf2 Signal

We hypothesized that Nrf2-mediated CH's antioxidation function against HG-induced HUVEC injury. To verify this hypothesis, Nrf2 activator bardoxolone and Nrf2 inhibitor ML385 were used, respectively, to regulate Nrf2 signaling activation.

**Figure 8:**
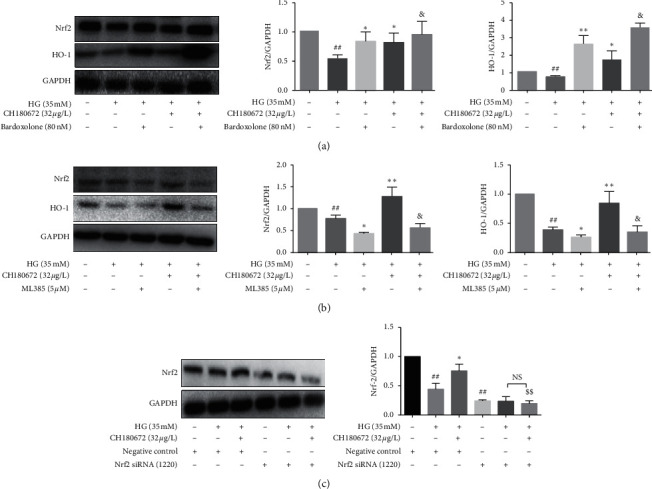
CH increased the Nrf2 protein level in HG-induced HUVECs. (a) Cells were pretreated with/without 80 nM bardoxolone for 1.5 h, followed by incubating with/without 32 *μ*M CH for 24 h Western blot analysis of Nrf2 protein levels after treatment. (b) Cells were pretreated with/without 5 *µ*M ML385 for 1.5 h, followed by incubating with/without 32 *μ*M CH for 24 h. Western blot analysis of Nrf2 protein levels after treatment. (c) Using siRNA technology to silent Nrf2. After 24 hours of treatment, Nrf2 was knocked down by siRNA (siRNA-Nrf2), and then, these cells were exposed to normal medium for 24 h, respectively. A disordered sequence siRNA (siRNA-NC) was transfected into HUVECs, and then, these cells were exposed to normal medium for 24 h. Results were represented as the mean ± SEM (*n* = 3). ^#^*P* < 0.05 vs. control group; ^##^*P* < 0.01 vs. control group; ^*∗*^*P* < 0.05 vs. model group; ^&^*P* > 0.05 vs. HG + bardoxolone or HG + ML385 group; ^$$^*P* < 0.01 vs. HG + CH + NC group; ^N.S^*P* > 0.05 vs. HG + siNrf2 group.

Western blot analysis revealed that Nrf2 and HO-1 protein were significantly downregulated when treated with HG (35 mM) as compared to the control group (*P* < 0.01). When HUVECs were pretreated with CH (32 *μ*g/L) + bardoxolone (80 nM), we found that Nrf2 and HO-1 protein were upregulated compared with the HG group (Figure 8(a)). However, ML385 abrogated the improvement of CH on Nrf2 and HO-1 protein expression (Figure 8(b)). These results suggested that the protection effect of CH on endothelial cells was depended on the Nrf2/HO-1 signaling activation.

We demonstrated that CH can significantly upregulate Nrf2 expression to attenuate endothelial dysfunction. Moreover, we found that the activator of Nrf2 enhanced the protective function of CH. Meanwhile, we also found that the inhibitor of Nrf2 abolished the protective function of CH. Further evidence from either pharmacological agonist or inhibition revealed that the protective effect of CH on endothelial injury induced by HG occurred through the Nrf2/HO-1 signal pathway.

As shown in Figure 8(a), the experiment used siRNA technology to transfect HUVECs to achieve low expression of Nrf2, and the Nrf2 knockdown efficiency was about 65%. Compared with the HG group, the expression of Nrf2 protein in HUVECs pretreated with CH (32 *μ*g/L) was significantly upregulated. When the expression of Nrf2 was downregulated by siRNA, the role of CH in regulating Nrf2 was weakened. It can be seen from the experimental results that CH improves HG-induced HUVECs damage based on the Nrf2 signaling pathway.

### 3.9. Identification of the UHPLC-MS Structure of CH180672 Extract

**Figure 9:**
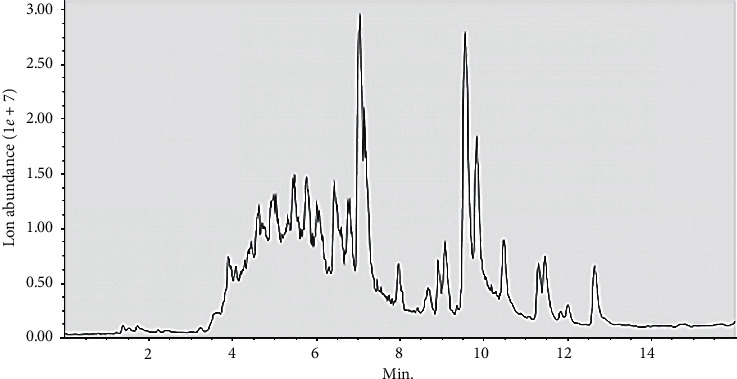
CH180672 extract anion chromatogram.

First, it can be judged from the negative ion peak diagram that the CH180672 extract contains compounds (Figure 9), and then the raw data of UPLHC-MS are imported into MS-DIAL 3.82 (MS-DIAL: data-independent MS/MS deconvolution for comprehensive metabolome analysis. 523–526, 2015). The software performs preprocessing, including peak extraction, denoising, deconvolution, peak alignment, and export of a 3D data matrix (raw data matrix) in CSV format. The extracted peak information is compared with the database MassBank, Respect, GNPS (14951 records in total) three libraries for full library search. This three-dimensional matrix includes information including sample information, retention time, mass-to-nuclear ratio, and mass spectrum response intensity (peak area). From the results, it was preliminarily judged that CH180672 ethyl acetate extract contained chemical components such as adenine, 2′-deoxyadenosine, adenosine, uracil, paeonol, and pilocarpine (Table 1). Studies have shown that adenosine is an endogenous nucleoside with a short half-life, which can regulate the physiological function of the heart and cardiovascular system [25]. In addition, paeonol can prevent tunicamycin-induced vascular endothelial dysfunction by inhibiting endoplasmic reticulum stress and oxidative stress [26]. Chen et al. pointed out that ginsenoside-Rg1 reduces D-galactose-induced oxidative stress in mice and increases the activity of superoxide dismutase and glutathione peroxidase in *vivo* [27]. Another study showed that magnolol reduced TNF-*α*-induced vascular endothelial cell damage [28]. Therefore, the components identified in the CH extract have a certain protective effect on vascular endothelial cell damage, which lays a material foundation for its pharmacological activity.

## 4. Conclusion

In summary, it is the first time to confirm that CH can inhibit HG-induced HUVEC oxidative damage and reveals the potential mechanism involved in the Nrf2/HO-1 signaling pathway and the protective effect of insect fungal extracts of *Simplicillium* sp. CH inhibits the oxidative damage of HUVECs induced by HG and provides a pharmacological basis for the prevention and treatment of diabetic complications. In addition, it was preliminarily determined that CH180672 ethyl acetate extract contained chemical components, such as adenine, 2′-deoxyadenosine, adenosine, uracil, paeonol, and pilocarpine based on UPLHC-MS results. This provides a certain material basis for the pharmacological effect of CH.

## Figures and Tables

**Table 1:** CH180672 UHPLC-mass structure identification table.

Compounds	Area	*m*/*z* value	Adduction	MS1 isotopes
Adenine	6719554	136.0636	[M + H]^+^	117.6775
2′-Deoxyadenosine	1039730	252.1101	[M + H]^+^	251.62872
Adenosine	1183380	268.106	[M + H]^+^	269.0701
Nicotinamide	768809	123.0558	[M + H]^+^	124.08461
Uracil	298905.1	113.035	[M + H]^+^	114.0512
Thymine	139144	127.0407	[M + H]^+^	128.09082
Sinomenine	120805.6	330.1759	[M + H]^+^	332.19388
Paeonol	996976.2	167.0729	[M + H]^+^	168.78964
Pilocarpine	237427.4	209.1547	[M + H]^+^	208.88545
Ginsenoside Rg1	14236.68	845.5034	[M + FA − H]^−^	845.69629
Magnolol	78533.37	265.1191	[M − H]^−^	264.99506
Ginsenoside Rh2 (S-FORM)	47211.7	667.426	[M + FA− H]^−^	68.83405

## Data Availability

All data used to support the findings of this study are included within the article.
